# Inhibition effect of *Bifidobacterium longum*, *Lactobacillus acidophilus*, *Streptococcus thermophilus* and *Enterococcus faecalis* and their related products on human colonic smooth muscle *in vitro*

**DOI:** 10.1371/journal.pone.0189257

**Published:** 2017-12-07

**Authors:** Jing Gong, Tao Bai, Lei Zhang, Wei Qian, Jun Song, Xiaohua Hou

**Affiliations:** Division of Gastroenterology, Union Hospital, Tongji Medical College, Huazhong University of Science and Technology, Wuhan, China; University of Texas Medical Branch, UNITED STATES

## Abstract

**Objective:**

To investigate the effects of four strains, generally used in clinic, including *Bifidobacterium longum*, *Lactobacillus acidophilus*, *Streptococcus thermophilus* and *Enterococcus faecalis*, and their related products on human colonic smooth muscle *in vitro*.

**Methods:**

Human colonic circular muscle strips obtained from disease-free margins of resected segments from 25 patients with colorectal cancer were isometrically examined in a constant-temperature organ bath and exposed to different concentrations of living bacteria, sonicated cell fractions and cell-free supernatant (CFS). The area under the curve (AUC) representing the contractility of smooth muscle strips was calculated.

**Results:**

(1) The four living probiotics inhibited the contractility of human colonic muscle strips only at high concentration (10^10^ CFUs/mL, all *P*<0.05). (2) The sonicated cell fractions from the four probiotics obviously inhibited human colonic smooth muscle strips in a dose-dependent manner (*P*<0.01). (3) The CFS from the four probiotics also inhibited colonic smooth muscle strips in a dose-dependent manner (all *P*<0.05). (4) The inhibition effect of CFS from *Streptococcus thermophilus* and *Enterococcus faecalis* decreased obviously when pretreated with NG-nitro-L-arginine (L-NNA, 10^−5^ mol/L) (*P*<0.05), but not the *Bifidobacterium longum* and *Lactobacillus acidophilus* (*P*>0.05).

**Conclusion:**

Four common probiotics related products, including the sonicated cell fractions and the CFS, obviously inhibited human colonic smooth muscles strips contraction in a dose-dependent manner. Only high concentration living probiotics (10^10^ CFUs/mL) can inhibit the colonic smooth muscles strips contraction. The NO pathway may be partly involved in the inhibitory effect of CFS from *Streptococcus thermophilus* and *Enterococcus faecalis*.

## Introduction

Gastrointestinal (GI) dysmotility is common in GI disorders and is responsible for related symptoms such as abdominal pain and distension [1]. Gut microbiota play an important role in GI motility regulation [2], which are related with intestinal transit [3,4]. The richness and composition of gut microbiota are strongly associated with stool consistency, which is a proxy for colonic transit rate [5]. In a recent study, the antibiotic-induced depletion of murine microbiota delayed the whole gut and colonic transit and reduced spontaneous contractions and the response to acetylcholine in the ileum and colon smooth muscle [6]. Manipulating gut microbiota might influence GI motility and further improve GI motility disorders [7–10].

According to preclinical and clinical studies, probiotics regulate GI motility by modulating the gut microbiota [11]. *Lactobacillus paracasei* obviously attenuated muscle hypercontractility in a murine model of post-infective gut dysfunction [12], and probiotic preparation VSL#3 (e.g., *Bifidobacterium*, *Lactobacillus*, *Streptococcus*) could modulate the intestinal smooth muscle activity [13]. Moreover, *Lactobacillus rhamnosus* and its supernatants reduced the lipopolysaccharide- induced inhibition of human colonic smooth muscle strip contractile response [14]. However, the strain, dose and intervention time were different in previous studies.

At present, the effects of probiotics on intestinal motility are mainly from clinical observation, but there is no strong evidence for the direct actions of probiotics on intestinal motor activity [15]. Whether probiotics themselves or their metabolic products affect GI smooth muscle remains unknown. Most of the previous studies have focused on the influence of probiotics on animal GI smooth muscle; few studies have investigated the effect of probiotics on human intestinal smooth muscle. Different probiotics might play different roles in GI motility. Most probiotics in clinical use are generally selected from the *Lactobacillus* and *Bifidobacterium* genera (which are common in the gut and are also used in probiotics drugs), *Escherichia coli* (the most common additive in yogurt, which is extensively enjoyed as probiotic-containing food) and *Enterococcus faecalis* strains (an opportunistic pathogen widely present in the intestine and supplied as a probiotic in some drugs with complex beneficial microorganisms).[16,17] Although these probiotics improve GI diseases, such as IBS, little is known about their effect on human smooth muscle contraction.[18]

Thus, the aim of this study was to investigate the effect of four probiotics commonly used clinically, including *Bifidobacterium longum* (*B*. *longum*), *Lactobacillus acidophilus* (*L*. *acidophilus*), *Streptococcus thermophilus* (*S*. *thermophilus*) and *Enterococcus faecalis* (*E*. *faecalis*), and their related products on human intestinal smooth muscle contraction and further compare the different effects on smooth muscle contraction. Additionally, we also investigated the possible role of the nitric oxide (NO) pathway in this process. Our study will identify the possible roles of specific probiotic strains in human intestinal motility.

## Materials and methods

### Bacterial preparation

#### Probiotics freeze-dried powder

Viable bacterial strains of *B*. *longum* HB55020 (1.6×10^12^ CFUs/g), *L*. *acidophilus* HB56003 (2.15×10^11^ CFUs/g), *S*. *thermophilus* HB5621 (2.78×10^12^ CFUs/g) and *E*. *faecalis* HB62001 (3.81×10^12^ CFUs/g) were obtained from Hubei Center of Industrial Culture Collection and Research (HBCC). Each strain was converted to a freeze-dried powder that was mixed with glucose and stored at -20°C for further use. The viable bacterial count was calculated by culture and colony counting method after dilution.

#### Cell-free supernatant (CFS) preparation

The method used to obtain CFS was described elsewhere [19]. Simply, the isolated probiotic strains *B*. *longum* HB55020, *L*. *acidophilus* HB56003, *S*. *thermophilus* HB5621 and *E*. *faecalis* HB62001 were cultured anaerobically in De Man-Rogosa-Sharpe (MRS) medium until plateau. The respective CFS was obtained by centrifugation at 12,000×g for 10 min, adjusted with 1 M NaOH to a pH of 7.0, and filtered through a 0.22-mm filter. The CFS was stored at -20°C for further use.

#### Preparation of bacterial cytoplasm crude extracts

The method was performed according to a previous study [20]. The freeze-dried powders of isolated probiotic strains *B*. *longum* HB55020, *L*. *acidophilus* HB56003, *S*. *thermophilus* HB5621 and *E*. *faecalis* HB62001 were resuspended in sterile PBS, and the final bacterial concentration was 10^9^−10^10^ colony forming units (CFUs/mL). The bacterial suspensions were sonicated at power level 8 in an ice bath. The duty time and interval time were 45 s and 15 s, respectively. There were 30 cycles, and the total time was 30 min. After ultrasonic dispersion, the bacterial suspensions were centrifuged at 4000×g for 10 min, and the CFS containing the probiotic crude extracts was collected.

### Patients

Twenty-five patients suffering from colonic carcinoma and undergoing surgery treatment were recruited from the GI Surgery Department at Union Hospital of Tongji Medical College of Huazhong University of Science and Technology. Exclusion criteria included no IBD, IBS or other functional GI disease; no hyperthyroidism; no diabetes mellitus or other chronic disease; no history of abdominal operation or trauma; no history of drug use; and no radiotherapy or chemotherapy before operation. The study protocol was approved by the Ethics Committee of Tongji Medical College, Huazhong University of Science and Technology (2010(72)).

### Tissue preparation

Human colon specimens were obtained from disease-free margins of resected segments from the colons of patients with colon cancer. Normal colorectal tissue at least 5 cm from the carcinoma lesion was obtained after the operation. Fresh specimens were placed in oxygenated (95% O_2_+5% CO_2_) Krebs solution. After cutting the bowel along the mesentery and removing the mucosa and submucosa layer, a smooth muscle strip approximately 3×10 mm from circular layer was made by a handmade double blade. Then, the muscle strip was ligated at each end by a silk suture.

### Recording of muscle strip tension

The method used to record muscle strip tension was described elsewhere [13,21]. In brief, the prepared muscle strip was suspended in a 37°C constant-temperature tissue bath containing 25 mL of oxygenated Krebs solution. The inferior extremity of the muscle strip was fixed with a hanger, and the superior extremity was connected to a tonotransducer (Fort-10, WPI company, American). The smooth muscle strip was balanced by 2 g of preload. Fresh Krebs solution was replaced every 20 min. The spontaneous contraction activity of the smooth muscle strip was stable for 1 h before any experimental procedures began.

### The effect of viable probiotics on human colonic smooth muscle strips from circular layer contraction

After stabilization for 1 h, the smooth muscle spontaneous activity was recorded as the baseline muscle strip contraction activity. Then viable *B*. *longum*, *L*. *acidophilus*, *S*. *thermophilus* or *E*. *faecalis* resuspensions with final concentrations (in the bath) at 10^7^ CFUs/mL, 10^8^ CFUs/mL, 10^9^ CFUs/mL and 10^10^ CFUs/mL were added cumulatively into the organ bath at intervals of 5 min, and the contractile curve was consecutively recorded. The changes in the smooth muscle strip contraction activity before and after viable probiotic administration was determined.

### The effect of crude probiotic extract on human colonic muscle strips from circular layer contraction

After stabilization for 1 h, the baseline smooth muscle spontaneous activity was recorded for 5 min. Then, the contraction activity of the muscle strips was recording for 5 min after the sequential addition of 10 μL, 50 μL, 100 μL, 200 μL and 300 μL of crude extract of different probiotics, respectively. The difference in the smooth muscle strip contraction activity before and after crude extract administration was determined.

### The influence of the cultured CFS of probiotics on human colonic smooth muscle strips from circular layer contraction

As previously described, the smooth muscle strips were stable for 60 min, and the baseline was recorded for 5 min. After sequentially adding 10 μL, 50 μL, 100 μL, 200 μL and 300 μL of the cultured probiotic CFS, respectively, the activity of the smooth muscle strips was recorded for 5 min. The control group included the addition of the same volume of MRS culture medium.

### The role of L-NNA in the effect of cultured CFS on human colonic smooth muscle strips from circular layer contraction

After spontaneous contraction stabilization for 60 min, the baseline was recorded for 5 min. L-NNA was added to the tissue bath at a final concentration of 10^−5^ mol/L, and the activity of the smooth muscle strip was recorded. Then, the cultured probiotic CFSs were added to the tissue bath at different concentrations as in the above mentioned protocol. The muscle strip activity was recorded for 5 min.

### Data processing

The area under the contractile curve (AUC) could be considered as comprehensive evaluation of the muscle activity containing the muscle tension, amplitude and frequency. The alteration of muscle strip contraction activity is represented by % inhibition of AUC to the control, which calculated as (effect value–control value)/control value. The effect value represents the AUC of the smooth muscle trip contraction after intervention, while the control value represents the AUC of the smooth muscle strip baseline contraction.

### Statistical analysis

The data are expressed with as the mean ± SE. GLM repeated measures analysis of variance (ANOVA) was applied to investigate the differences among the different groups. One-way ANOVA was performed using the least significant difference (LSD) of the homogeneity of variance; otherwise, Dunnett’s test was used. The paired *t* test was used to compare the differences between two groups, such as the inhibitory effects of *B*. *longum*, *L*. *acidophilus*, *S*. *thermophilus* or *E*. *faecalis* on the muscle activities with and without L-NNA pretreated. SPSS 18.0 (SPSS, Inc., Chicago, IL, USA) was used for all of the statistical analyses, and GraphPad Prism 5 software (GraphPad, San Diego, CA, USA) was used to create all of the related graphs. *P*<0.05 was considered statistically significant.

## Results

### Effects of living bacteria of human colonic smooth muscle contraction

**Fig 1:**
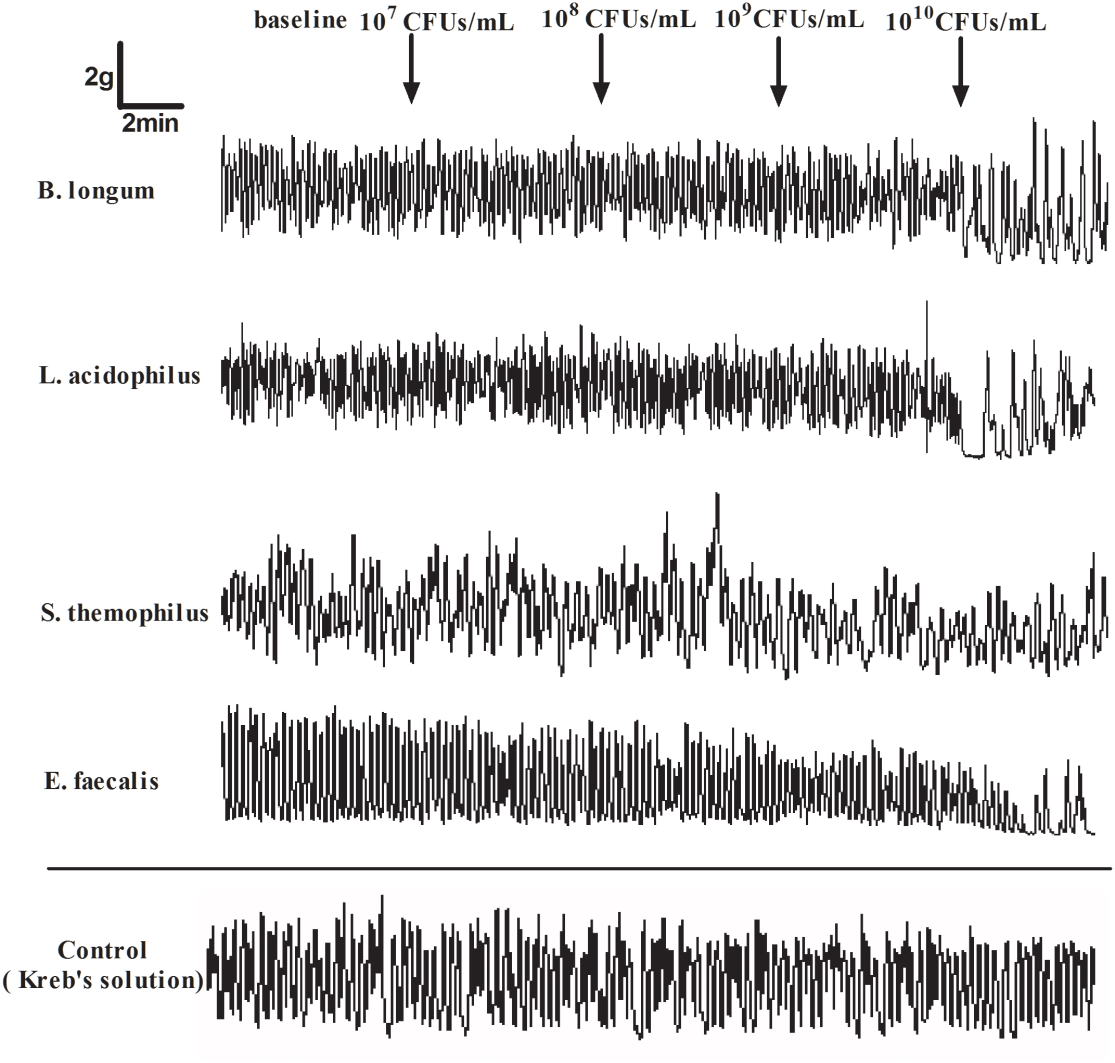
Typical human colonic smooth muscle contraction response to different viable probiotics at different concentrations. With higher concentrations (10^10^ CFUs/ml), human colonic smooth muscle contraction was obviously inhibited. The Kreb’s solution was used as vacuity control.

**Fig 2:**
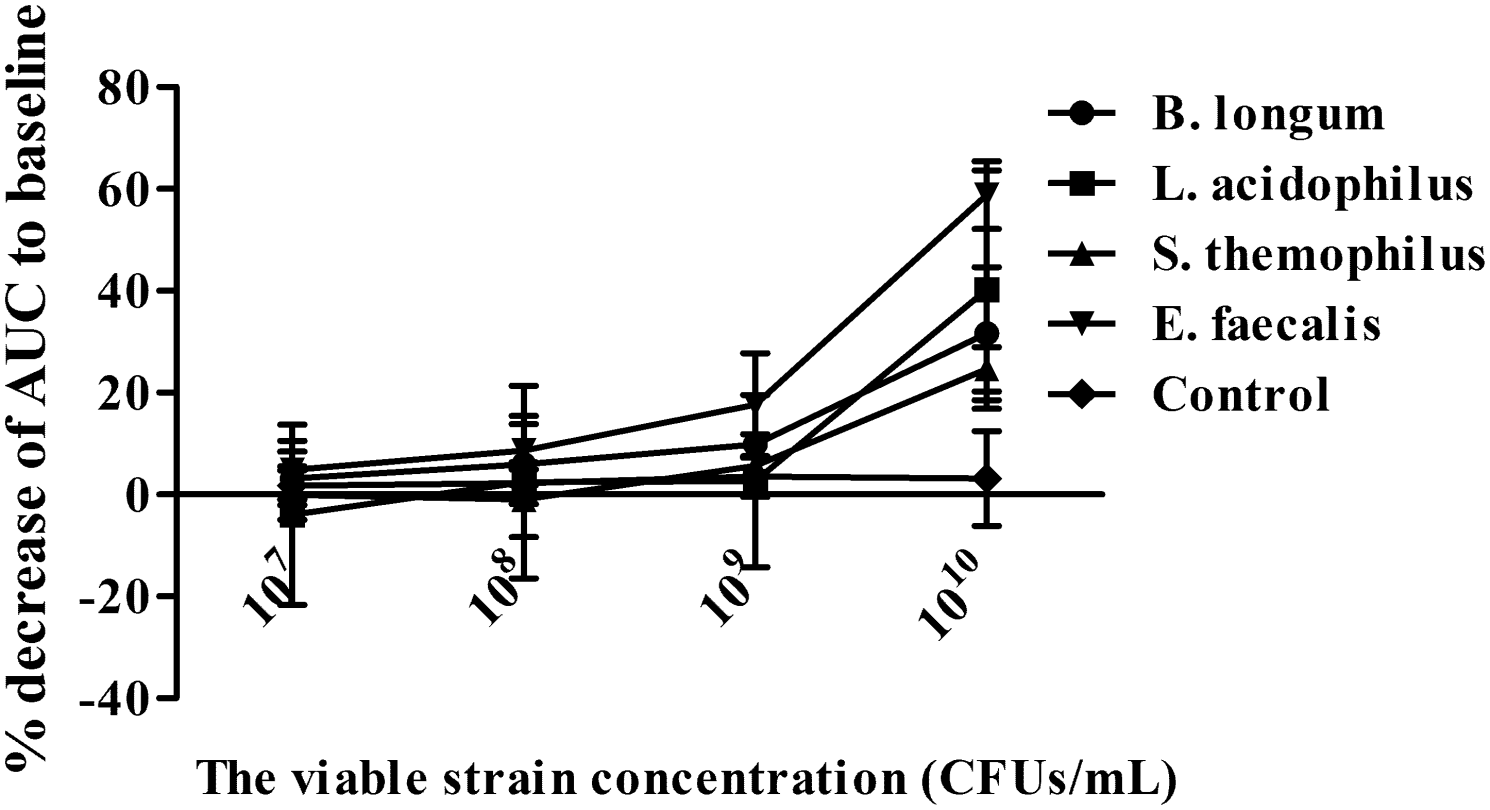
Living bacteria inhibited human colonic smooth muscle contraction. The higher concentration (10^10^ CFUs/mL) obviously inhibited human colonic smooth muscle contraction compared with lower contractions (all *P*<0.05). *E*. *faecalis* had the strongest inhibition compared with other probiotics (*P*<0.05). N = 6 for each group.

The human colonic smooth muscle contraction was not obviously changed after the addition of four viable probiotics at concentrations of 10^7^ to 10^9^ CFUs/mL, except for *E*. *faecalis* at 10^9^ CFUs/mL. Four viable probiotics at 10^10^ CFUs/mL obviously inhibited smooth muscle strip contraction *in vitro*. The % inhibition of AUC were 31.63±13.06, 40.24±23.40, 24.63±4.33 and 58.82±6.63 for *B*. *longum*, *L*. *acidophilus*, *S*. *thermophilus* and *E*. *faecalis* at 10^10^ CFUs/mL, respectively, which was significantly higher than that at 10^7^ CFUs/mL (all *P*<0.05). The inhibition effect of *E*. *faecalis* at 10^10^ CFUs/mL on muscle activity was strongest compared with that of the probiotics at the corresponding concentrations (*P*<0.05; Figs 1 and 2).Details was seen in S1 and S2 Files.

### Effects of crude extract of probiotics on human colonic smooth muscle contraction

**Fig 3:**
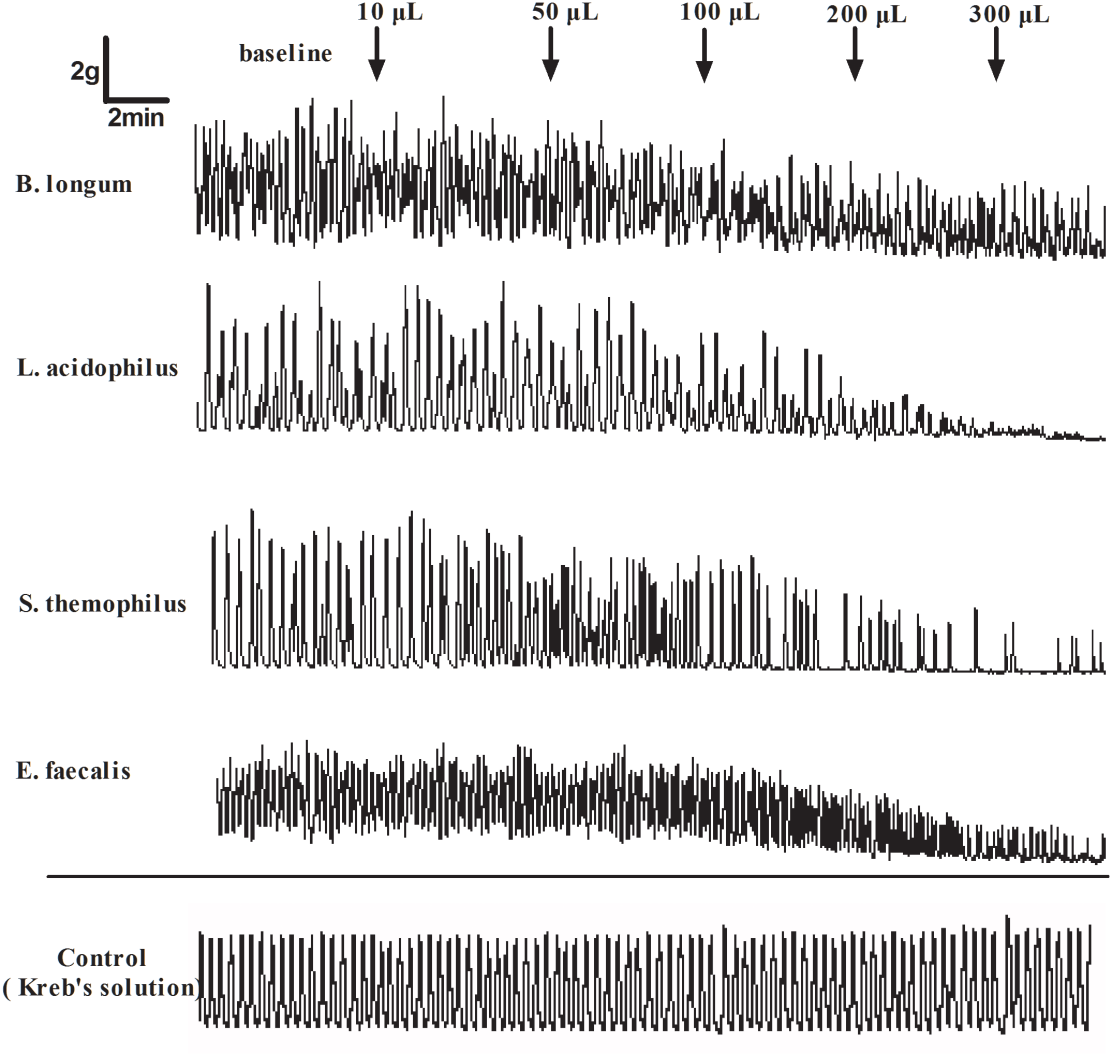
The typical human colonic smooth muscle contraction response to crude extracts. Higher crude extract concentrations produced a stronger inhibition of smooth muscle contraction. The Kreb’s solution was used as vacuity control.

**Fig 4:**
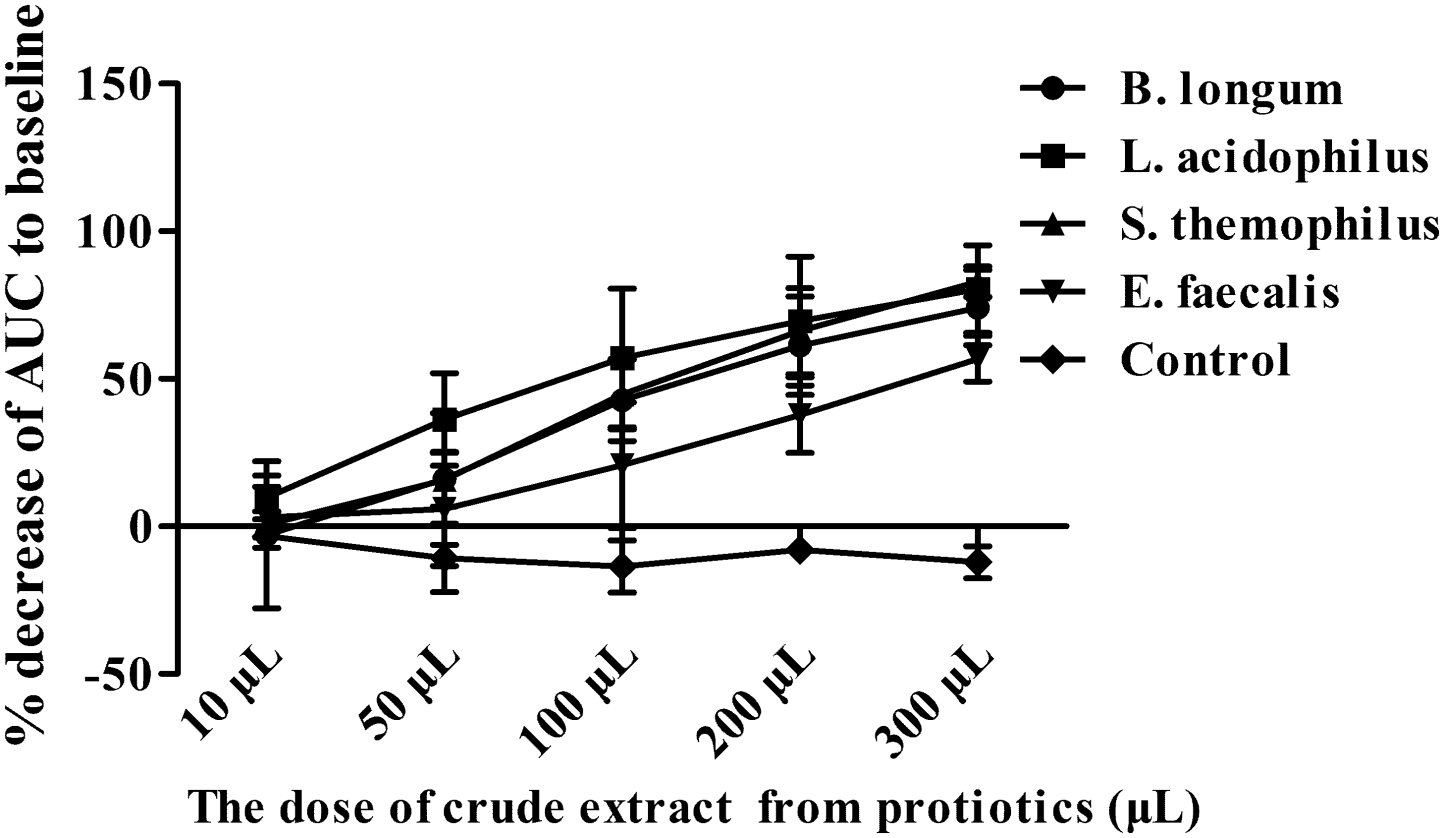
Crude extracts inhibited human colonic smooth muscle contraction in a dose-dependent manner. % inhibition of AUC increased with the increased concentration of crude extracts (all *P*<0.05). The inhibition effect of the crude extracts from *E*.*faecalis* was the weakest compared with others at 100μL, 200μL and 300μL dose (all p<0.05). N = 6 for each group.

The crude extracts from four types of probiotics obviously inhibited colonic circular muscle strip contraction *in vitro* in a dose-dependent manner. The strips were completely inhibited after adding 300 μL of crude extracts from different groups. The % inhibition of AUC induced by 300 μL of crude extracts was 74.15±12.74, 80.4±14.89, 82.88±5.15 and 56.81±7.33 from *B*. *longum*, *L*. *acidophilus*, *S*. *thermophilus* and *E*. *faecalis*, respectively, which was obviously higher than that induced by 10 μL of crude extracts (-2.84± 24.92, 9.78± 7.41, 0.64±4.42 and 3.06±10.38, respectively; all *P*<0.05). The inhibition effect of the crude extracts from *E*.*faecalis* on muscle activity was the weakest compared with others at 100μL, 200μL and 300μL dose (all p<0.05; Figs 3 and 4). Details was seen in S1 and S2 Files.

### Influence of CFS on human colonic smooth muscle activity

**Fig 5:**
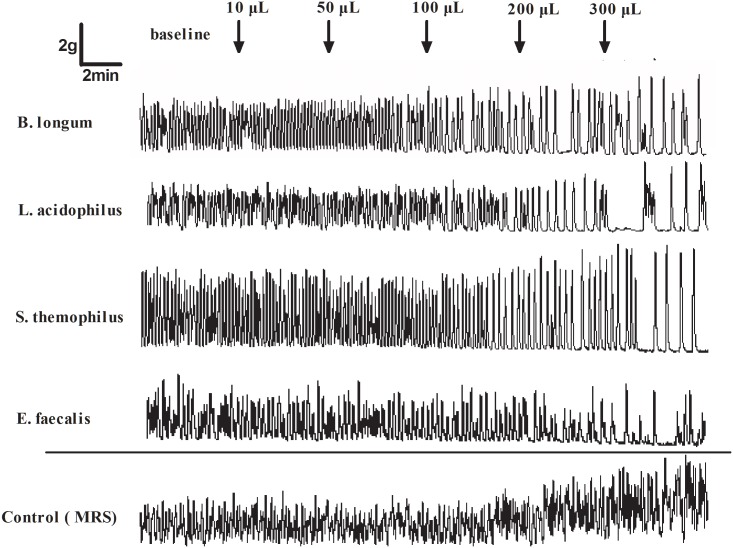
The typical human colonic smooth muscle contraction response to the sterile CFS. Higher CFS concentrations produced a stronger inhibition of smooth muscle contraction. The MRS (de *Man-Rogosa-Sharpe* broth-sodium nitrite medium) was used as vacuity control.

**Fig 6:**
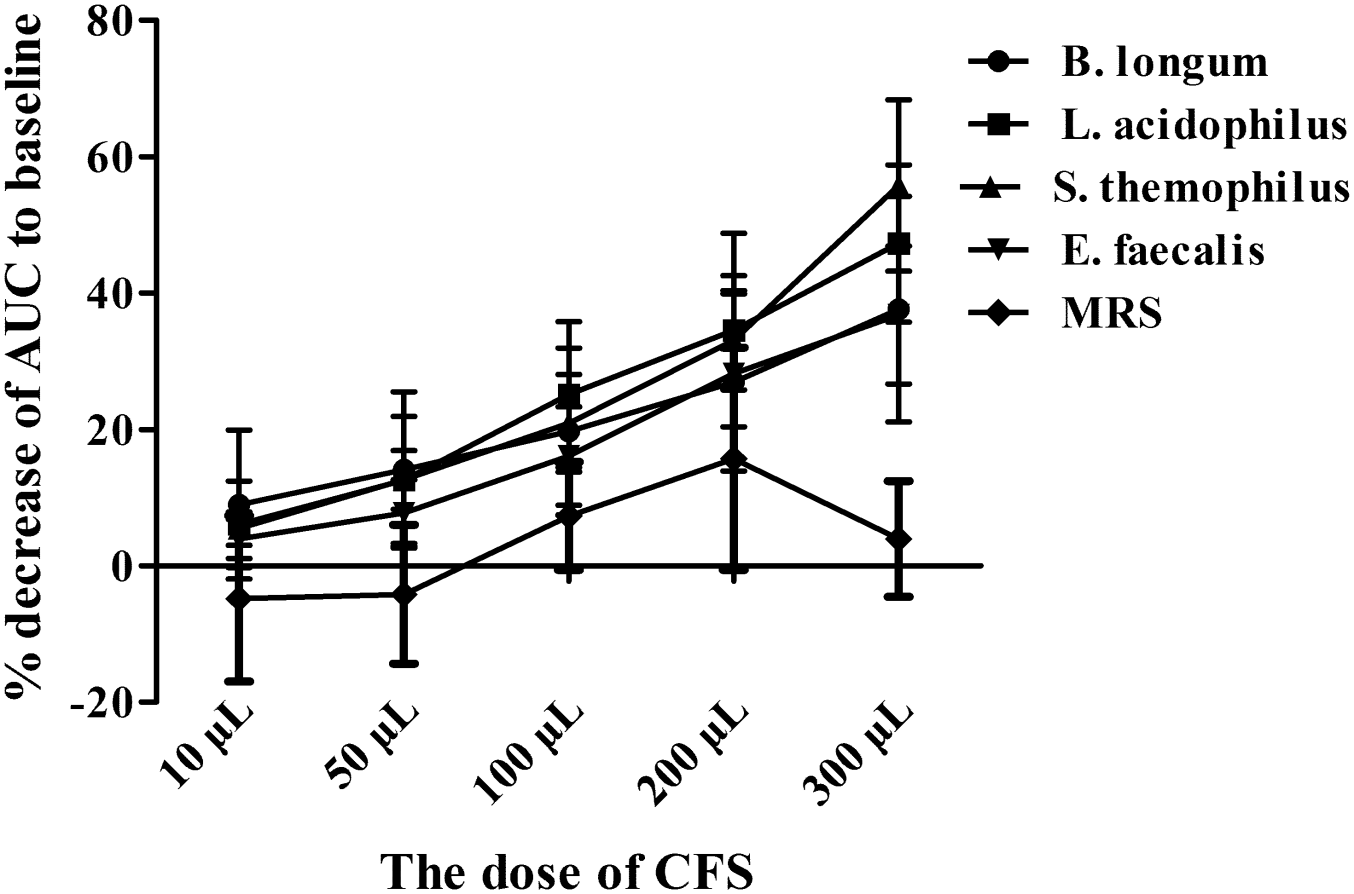
CFS inhibited human colonic smooth muscle contraction in a dose-dependent manner. % inhibition of AUC increased with the increased concentration of crude extracts except for at 10 μL (*P*<0.05). However, there was no obvious difference between the different probiotics (all *P*>0.05). N = 6 for each group. MRS, de *Man-Rogosa-Sharpe* broth-sodium nitrite medium.

The sterile CFS from four living probiotics obviously inhibited human colonic smooth muscle contraction in a concentration-dependent manner. The % inhibition of AUC induced by the CFS from four probiotics at different concentrations was significantly higher than that of control group except for the 10μL CFS group (all *P*<0.001). The AUC change rate induced by 300 μL of sterile CFS from *B*. *longum*, *L*. *acidophilus*, *S*. *thermophilus* and *E*. *faecalis* was 37.63±16.52, 47.27±11.54, 55.78±12.54 and 36.79±10.10, respectively, which was obviously higher than that induced by 10 μL of sterile CFS from the corresponding probiotics (8.98±10.91, 6.08±6.32, 5.46±2.46 and 3.94±2.89; all *P*<0.05). There was no obvious difference between the CFSs from different probiotics (Figs 5 and 6). Details was seen in S1 and S2 Files.

### Role of L-NNA in the action of CFS on human colonic smooth muscle contraction

**Fig 7:**
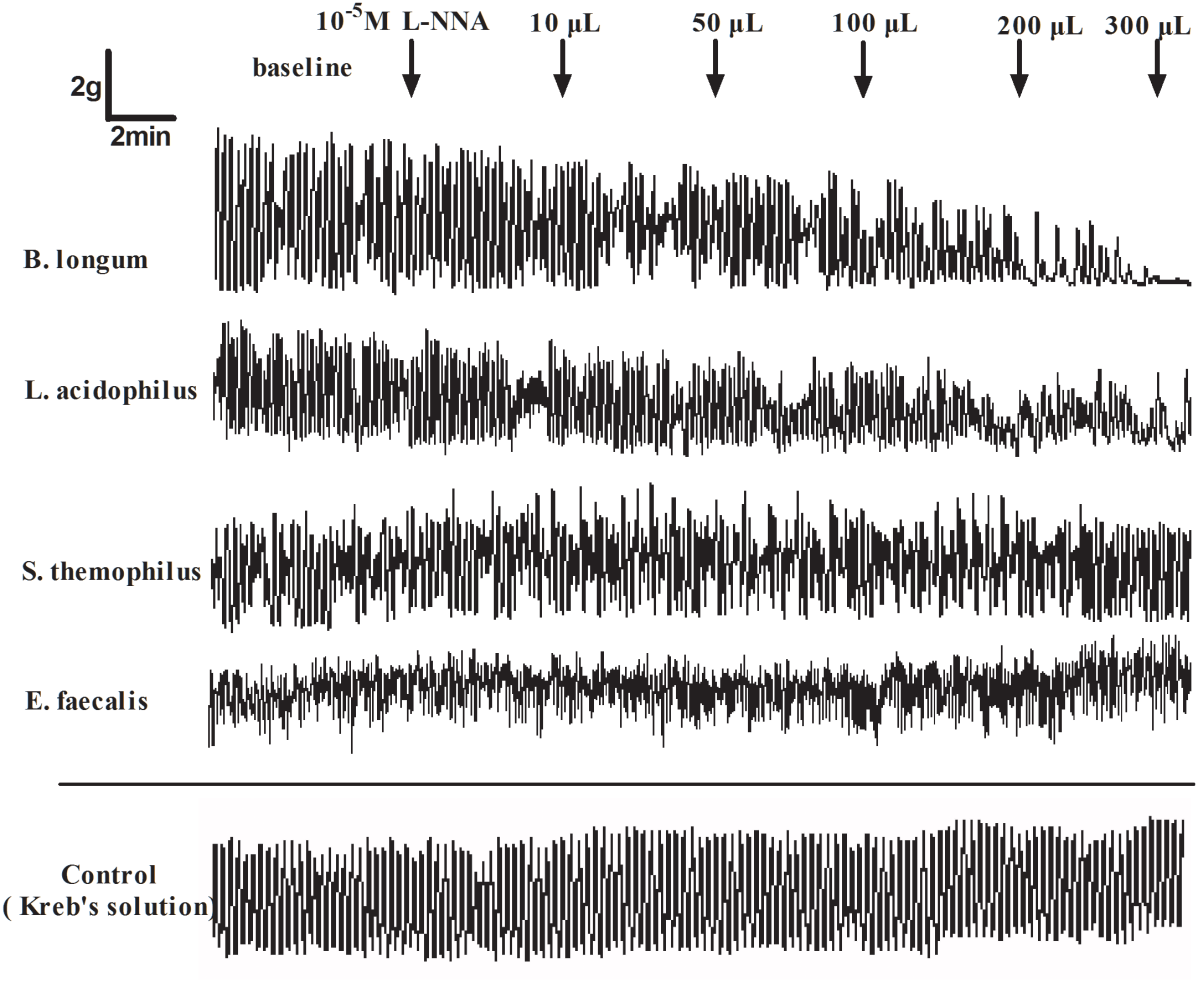
The typical human colonic smooth muscle contraction response to the sterile CFS after L-NNA pretreatment. The inhibition by the CFS from *S*. *thermophilus* and *E*. *faecalis* was obviously reversed after L-NNA intervention. The Kreb’s solution was used as vacuity control.

**Fig 8:**
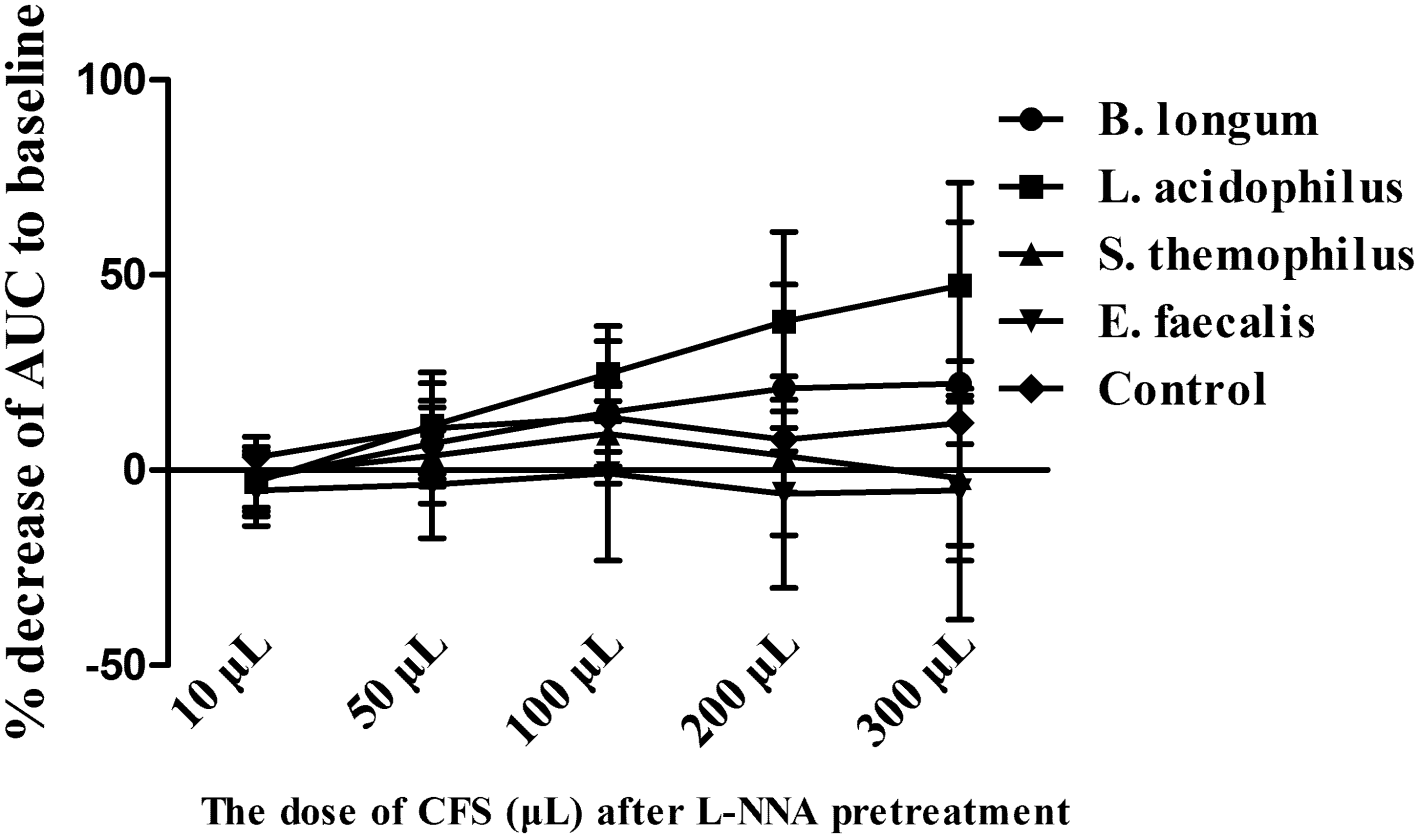
L-NNA pretreatment partly inhibited the response of human colonic smooth muscle to CFS. After L-NNA pretreatment, there was no obvious difference between various concentrations of CFS in *S*. *thermophilus* and *E*. *faecalis* (all *P*>0.05). However, there was no obvious effect of L-NNA pretreatment on the inhibition of *B*. *longum* and *L*. *acidophilus*. There was an obvious difference between the various concentrations of CFS in *B*. *longum* and *L*. *acidophilus* after pretreatment with L-NNA (all *P*<0.05). N = 6 for each group.

The administration of 10^−5^ mol/L L-NNA had no significant effect on human colonic smooth muscle strips compared with the baseline (AUC 615.86±85.14 vs. 600.52±48.40; *P*>0.05). However, the inhibition effect of different doses of CFS from *S*. *thermophilus* and *E*. *faecalis* on human colonic smooth muscle contraction was obviously reversed after L-NNA pretreatment. The % inhibition of AUC induced by 300 μL of sterile CFS from *S*. *thermophilus* and *E*. *faecalis* was 55.78±12.54 and 36.79±10.10, which was obviously higher than that when pretreated with 10^−5^ mol/L L-NNA (-2.03±21.13 and -5.18±33.08; all *P*<0.05). However, there was no obvious difference in the inhibition effect of 300 μL sterile CFS from *B*. *longum* and *L*. *acidophilus* when pretreated with L-NNA (37.63±16.52 *vs*.22.15±41.44; 47.27±11.54 *vs*. 47.32±36.37; *P*>0.05) (Figs 7 and 8). Details was seen in S1 and S2 Files.

## Discussion

This is the first study to investigate the direct effect of four strains, including *B*. *longum*, *L*. *acidophilus*, *S*. *thermophilus* and *E*. *faecalis*, and related metabolic products on human colonic smooth muscle contraction. The four living probiotics inhibited the contractility of human colonic muscle strips only at high concentration (10^10^ CFUs/mL). The crude extract and CFS from the four strains inhibited colonic smooth muscle strips in a dose-dependent manner. The NO pathway may be partly involved in the inhibitory effect of metabolic products of some strains on human colonic smooth muscle.

Probiotics are widely used to prevent and treat a variety of digestive diseases, however, the mechanism for the direct effect of probiotics on gut motility is not well known, especially for the effect on human colonic smooth muscle. It reported that *L*. *paracasei* attenuated muscle hypercontractility in a murine model of post-infective gut dysfunction infected with *Trichinella spiralis* [12]. It found that *L*. *rhamnosus* GG (LGG) shortened the human colon smooth muscle cell in a dose-dependent and time-dependent manner and inhibited the maximal Ach-induced human colon smooth muscle cell contraction [22]. Our study also found that four viable strains, including *B*. *longum*, *L*. *acidophilus*, *S*. *thermophilus* and *E*. *faecalis*, obviously and directly inhibited human smooth muscle contraction at 10^10^ CFUs/mL. Massi et al found that viable VSL#3 probiotic cocktail had no effect on guinea pig gut motility when different viable probiotics with 10^5^−10^9^ CFUs/mL stimulated guinea pig ileocolic smooth muscle strips [20], similar to our study. Only viable strains at a higher concentration inhibited smooth muscle contractions from the colonic circular layer. Our results and previous studies may partly explain the inefficiency of some probiotics in treating digestive diseases due to insufficient concentrations. Increasing the concentrations of probiotics in the colon may enhance their effectiveness.

However, a considerable body of evidence has confirmed that probiotics indeed improve the symptoms of GI diseases [23–27]. Thus, the aforementioned results also might suggest that the viable probiotics themselves are not responsible for inhibiting human colonic smooth muscle. The inhibitory role might be mediated by metabolites of the probiotics. Once the metabolites of the probiotics reach a certain threshold concentration, they may play roles in modulating GI motility. Indeed, our study found that both the crude extracts and CFS of the four strains inhibited the spontaneous contraction of human colonic smooth muscle strips from the circular layer *in vitro* in a dose-dependent manner. The higher concentrations of crude extracts and CFS from the four strains produced a stronger inhibition of human colonic smooth muscle strips. Massi et al also found that crude extracts of VSL#3 strains increased both spontaneous phasic and tonic contractions of the guinea-pig ileum in a dose-dependent manner [13]. Our human study was consistent with this animal observation. The human colonic smooth muscle strips in our study were dissected without mucosa and submucosa, which means that the altered contractile response of the smooth muscle strips was due to the direct influence of crude extracts and CFS from the four strains. Taken together, the metabolites of the four strains could directly inhibit the human colonic smooth muscle from the circular layer and further modulate the intestinal motility. The mechanisms of these processes require further investigation.

Another intriguing result of our study is the difference between the four strains in modulating human colon motility. *E*. *faecalis* had the strongest inhibition effect on human colonic circular smooth muscle strips compared to *B*. *longum*, *L*. *acidophilus*, and *S*. *thermophilus*. The inhibition of crude extracts from *L*. *acidophilus*, *S*. *thermophilus* and *B*. *longum* was stronger than that from *E*. *faecalis* at 300μL dose. Bär et al found that the CFS of *E*. *coli* Nissle 1917 enhanced the contractility of human colonic circular smooth muscle strips by directly stimulating smooth muscle cells [21]. These results are opposite those of our study. It reported the *L*. *paracasei* but not *L*. *johnsonii*, *B*. *lactis*, or *B*. *longum* attenuated muscle hypercontractility in a PI-IBS model infected with *Trichinella spiralis* [12]. It also found that crude extracts from different bacterial groups provoke different motility response patterns, i.e., *Bifidobacterium* and *Streptococcus* strains did not change the basal tone of the guinea pig ileum tissue, while *Lactobacillus* strains induced relevant guinea pig ileum contractions [13]. Ammoscato et al also found that the CFSs of *L*. *rhamnosus* GG significantly reduced the LPS-induced morpho-functional alterations of human colonic smooth muscle cells, such as cell shortening and contractile response inhibition [28]. The different effects of different strains on animal and human colonic strips means that the role of probiotics in intestinal motility differs between species. Probiotics showed strain-dependent modulation of intestinal motility, and different active metabolites, such as acetic acid, from various probiotics might be responsible to this distinction. Thus, the exact role of each strain and its metabolites in human GI motility require further investigation in order to guide clinical individualized treatment using probiotics.

Our study found that the NO synthase inhibitor, L-NNA, abolished the suppression effects of CFS from *S*. *thermophilus* and *E*. *faecalis* but not *B*. *longum* and *L*. *acidophilus*. The results indicate that NO is involved in the modulation by the metabolites of some stains on intestinal motility. Probiotics can affect the NO metabolism of the GI tract. Sobko et al found that dietary supplementation with *Lactobacilli* resulted in a 3- to 8-fold NO increase in the small intestine and caecum of rats. The NO levels in the colon correlated with the nitrite content of breast milk and feces from neonates, and *Lactobacilli* and *Bifidobacteria* isolated from the stools of neonates generated NO from nitrite *in vitro*. In contrast, *S*. *aureus* and *E*. *coli* were consumed NO rapidly. These results indicate that intestinal NO generation could be simulated by dietary supplementation with substrate and *Lactobacilli* [29]. In addition to affecting intestinal NO metabolism, probiotics also affect endogenous NO generation in the gut. For example, *L*. *rhamnosus* GG induced NO production in J774 macrophages and human T84 colon epithelial cells by inducing inducible NOS (iNOS) through a mechanism involving the activation of the transcription factor NF-kappaB pathway. The results indicated that the induction of iNOS and the low-level synthesis of NO may be involved in the protective actions of LGG in the GI tract [30]. The tissue used in our study did not contain mucosal constituents. The reversal by L-NNA of the inhibition by CFS from *S*. *thermophilus* and *E*. *faecalis* of human colonic smooth muscle strips indicated that the metabolites from some strains could induce NO production in other colonic tissues, such as smooth muscle cells and neuronal cells, but not epithelial cells through the induction of iNOS.

## Conclusions

Our study confirms that four probiotic strains and their metabolites directly modulate human colon motility *in vitro*, although the different strains played various roles. These roles are mainly mediated by the small-molecule metabolites or bacterial components of probiotics, which can cross the epithelial cell barrier. The mechanisms partly include the production of NO and require further research.

## Supporting information

S1 File The primary data for figures. (XLSX)

S2 File The primary recording for figures. (PDF)
