# Supplementary material for: Inhibition effect of Bifidobacterium longum, Lactobacillus acidophilus, Streptococcus thermophilus and Enterococcus faecalis and their related products on human colonic smooth muscle in vitro
Source: PLoS One. 2017 Dec 7;12(12):e0189257. doi: 10.1371/journal.pone.0189257 (PMC5720742; doi:10.1371/journal.pone.0189257)

Entire living bacteria of Bifidobacterium longum（with different concentration）

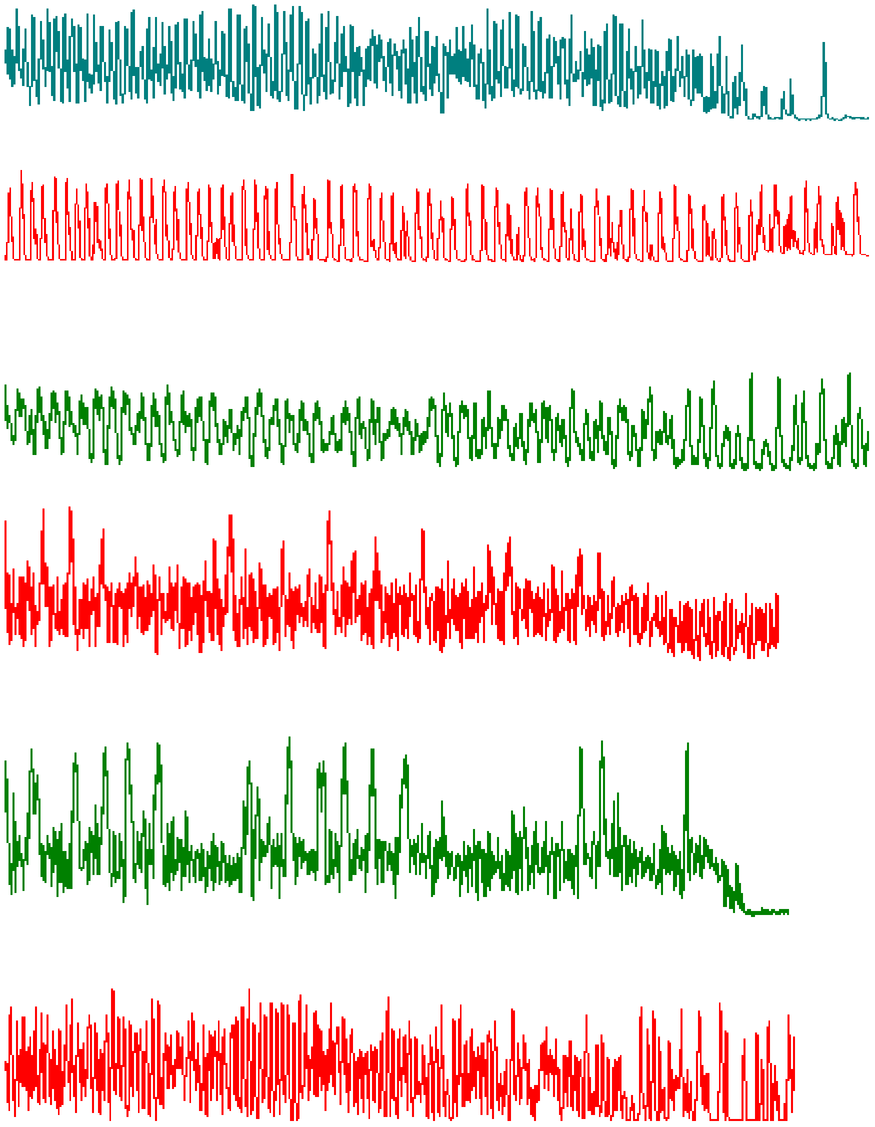

Cell-free supernatant of Bifidobacterium longum （with different dose）

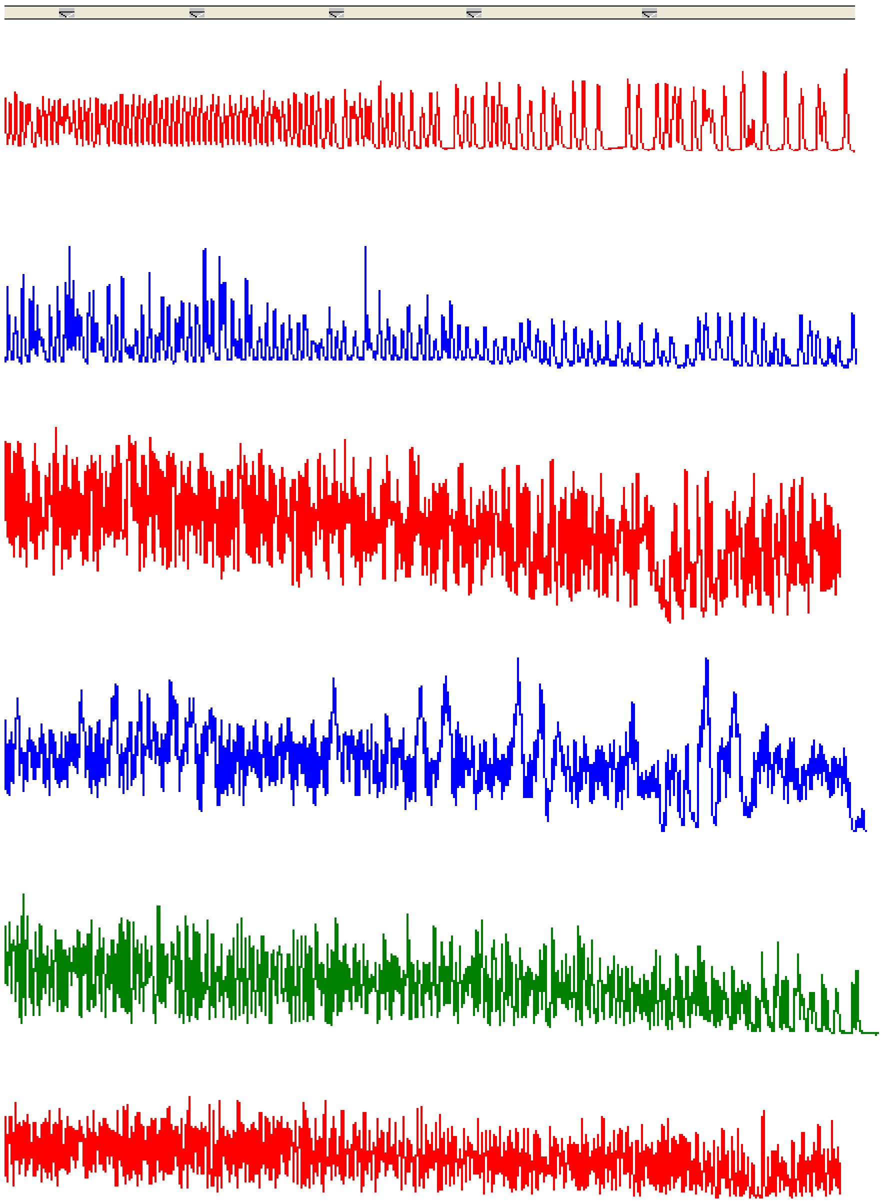

Sonicated cell fractions of Bifidobacterium longum （with different dose）

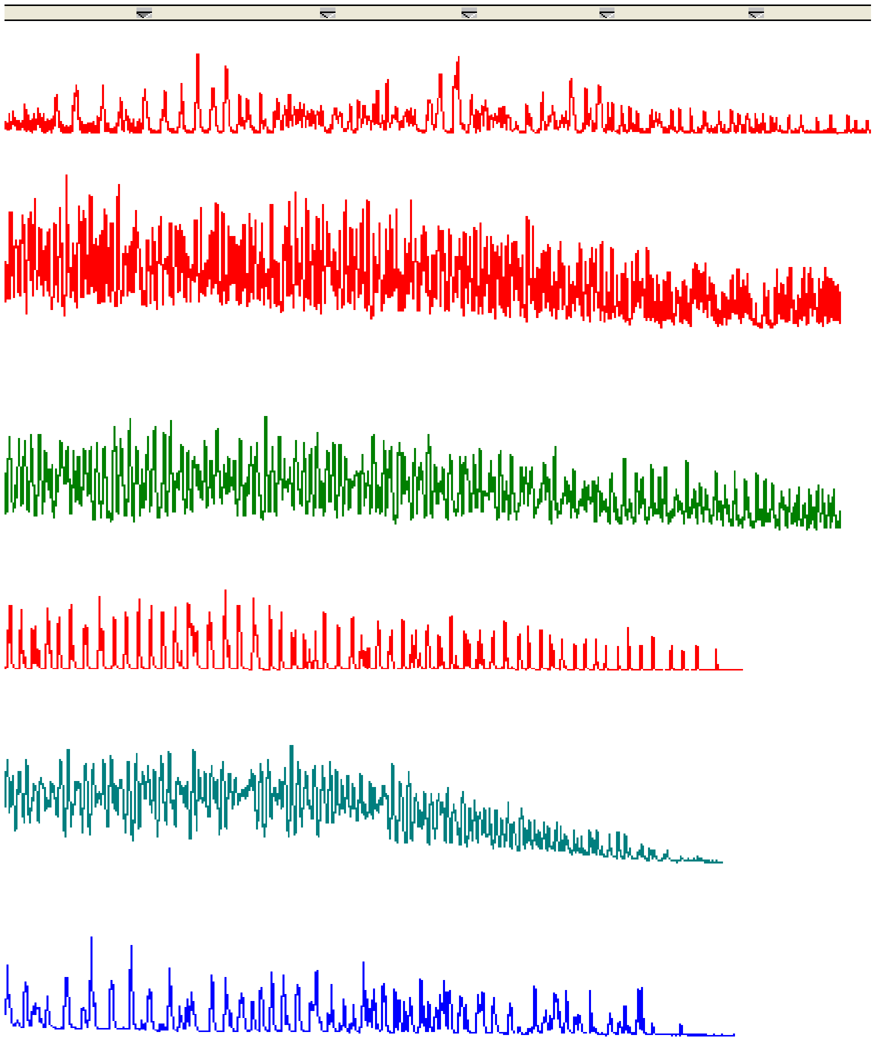

Entire living bacteria of Lactobacillus acidophilus （with different concentration）

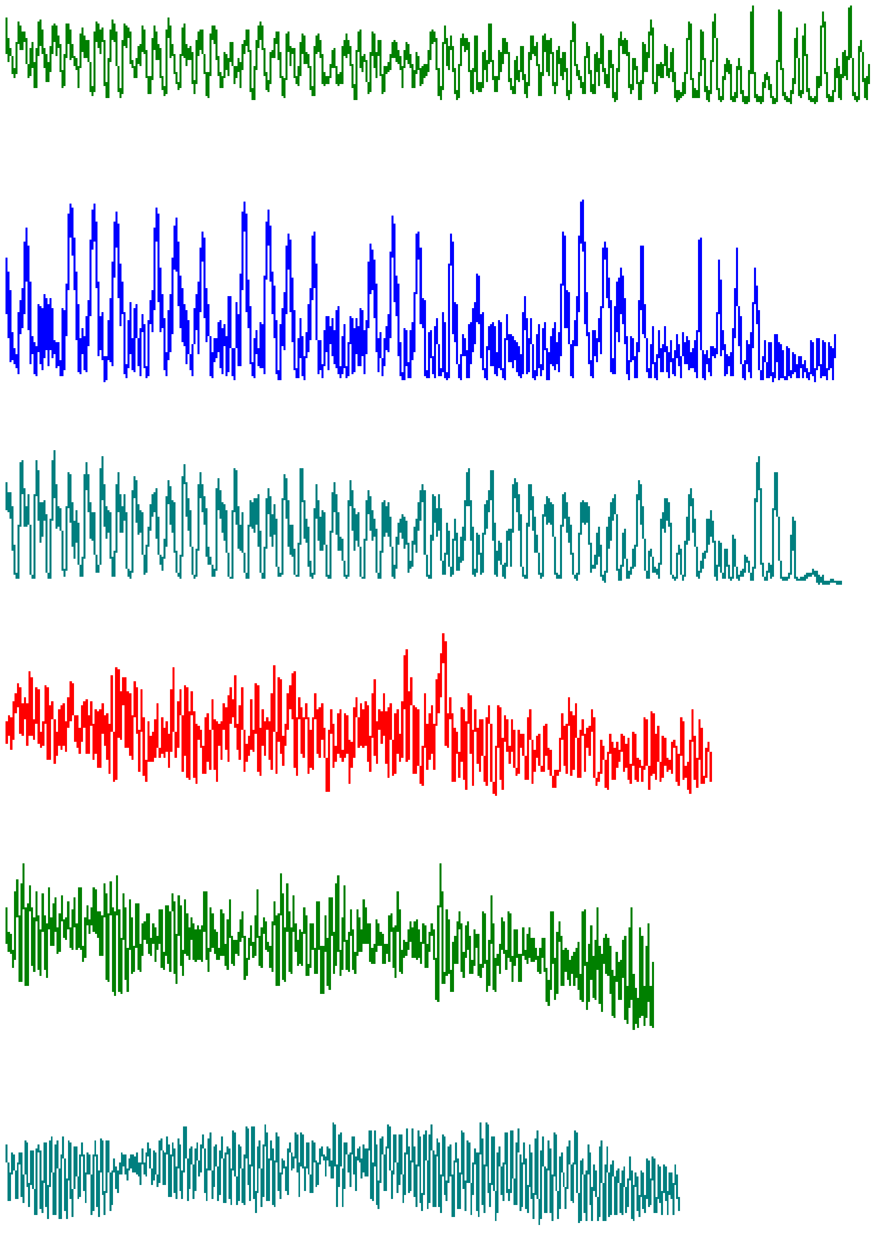

Cell-free supernatant of Lactobacillus acidophilus （with different dose）

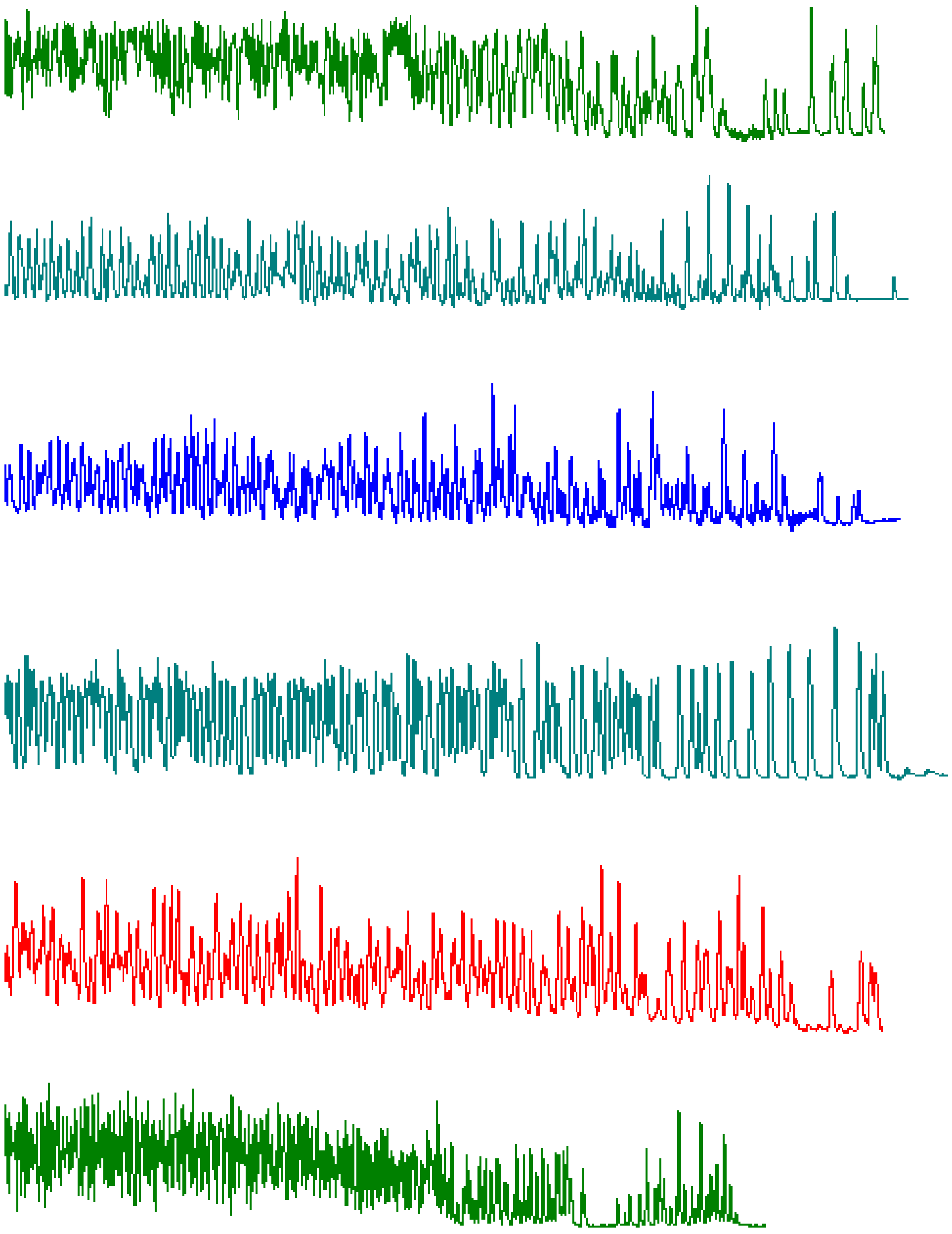

Crude extracts of Lactobacillus acidophilus（with different dose）

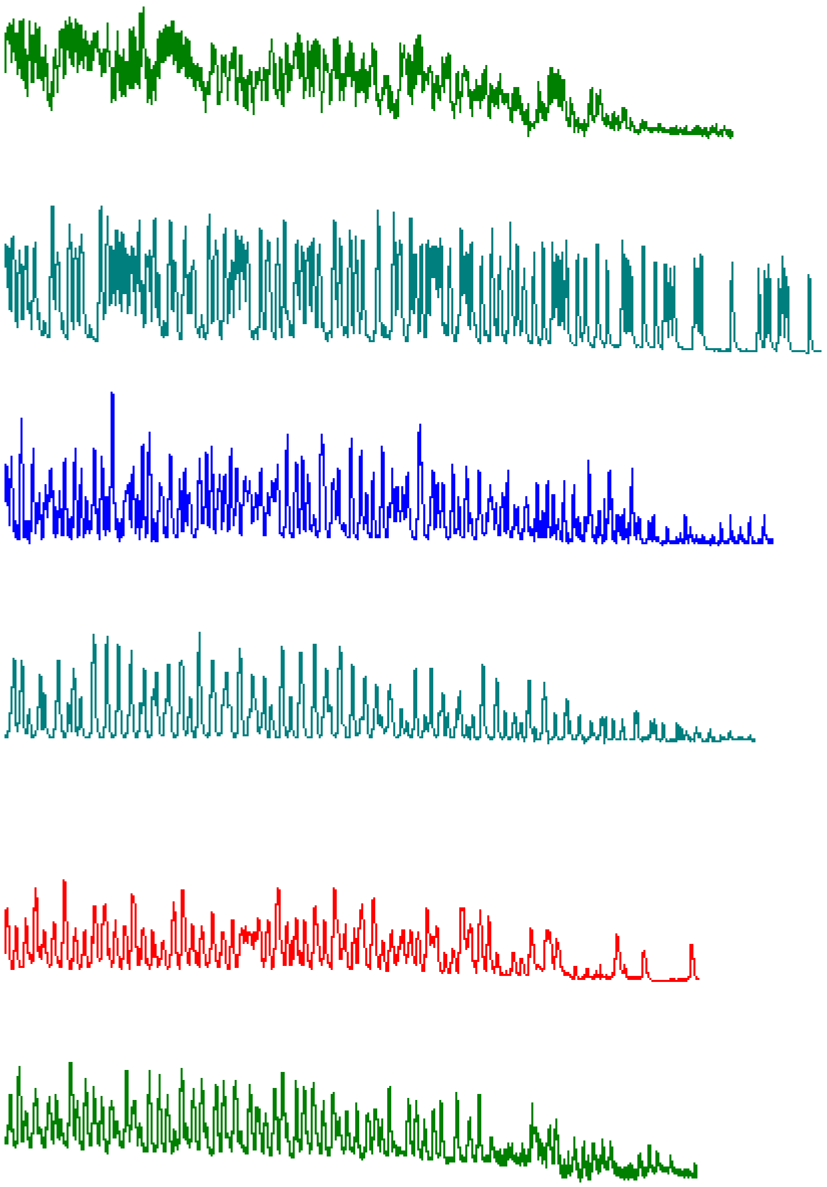

Entire living bacteria of Streptococcus themophilus （with different concentration）

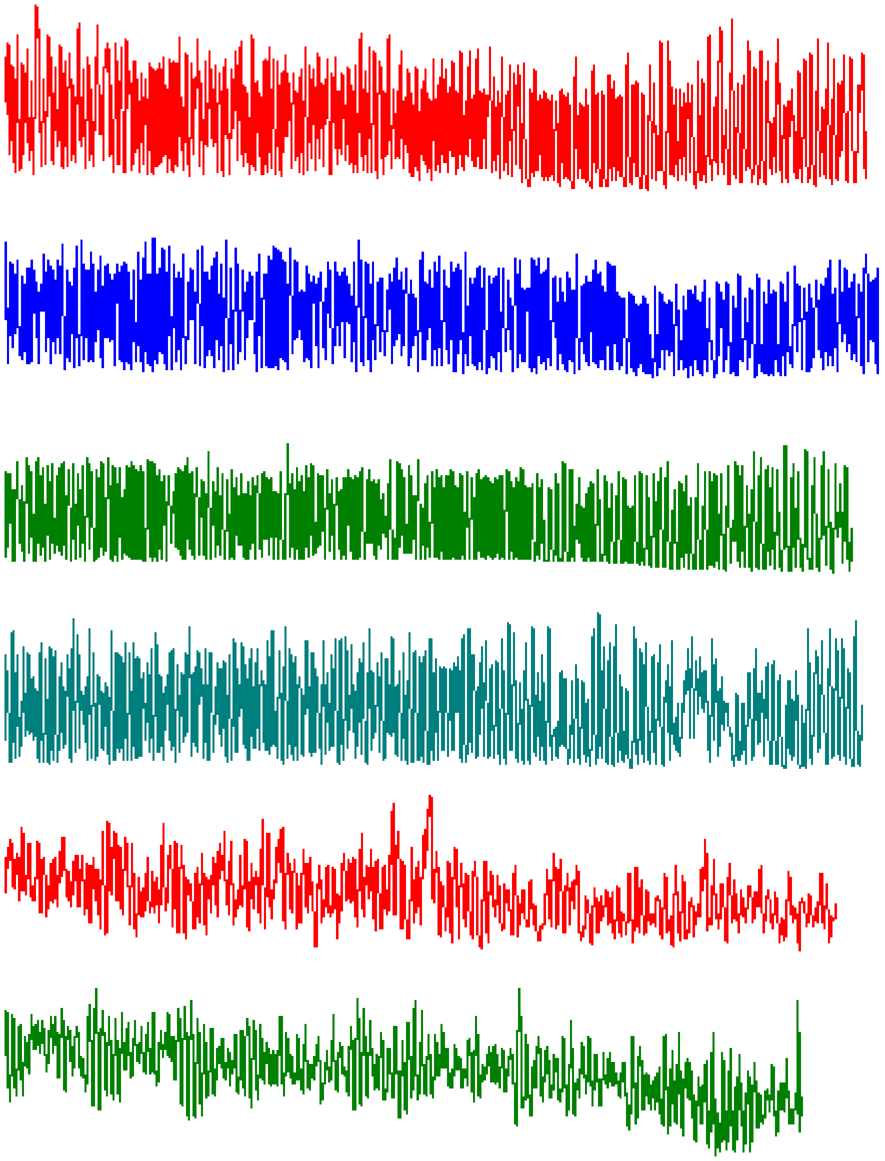

Cell-free supernatant of Streptococcus themophilus （with different dose）

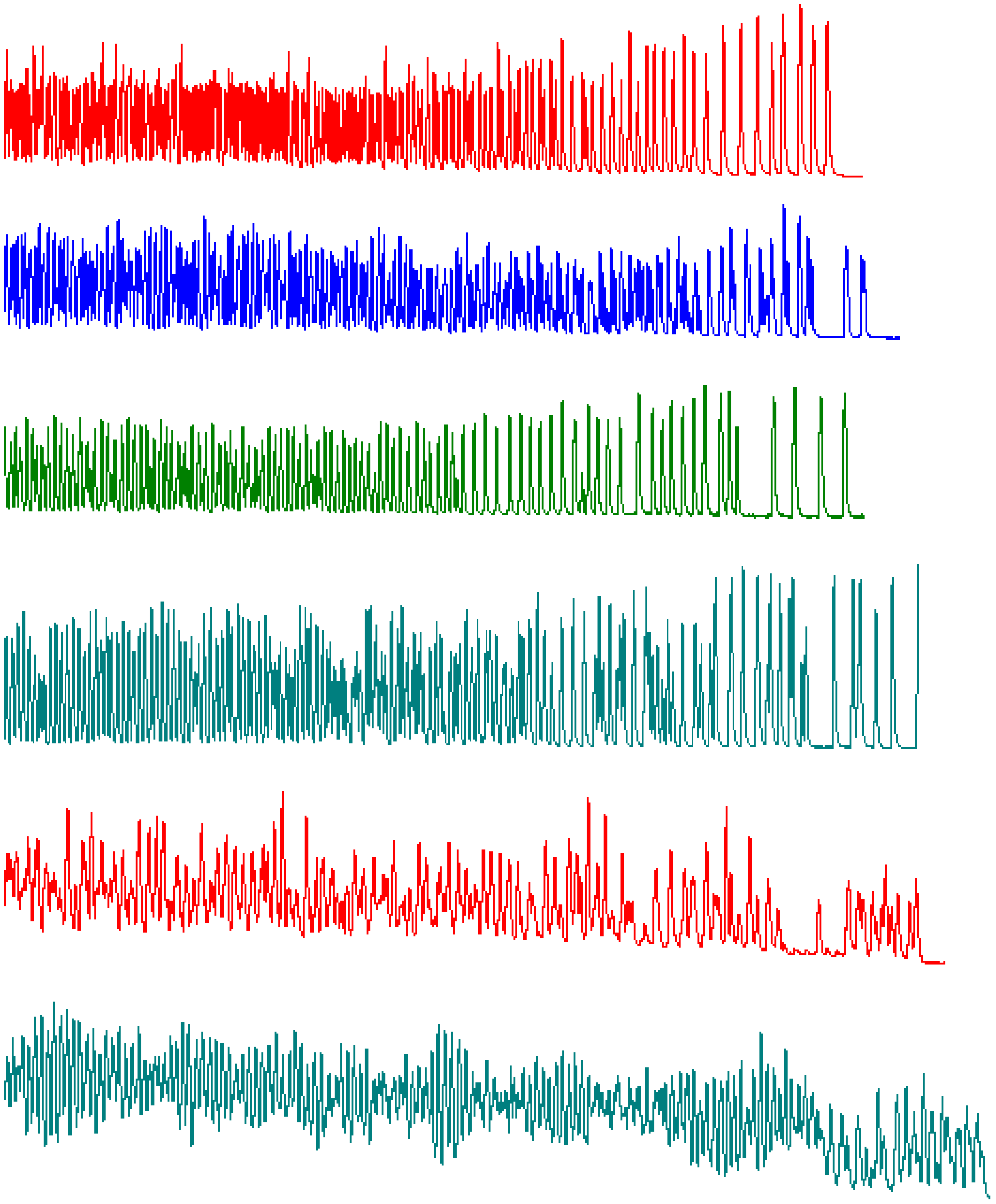

Crude extracts of Streptococcus themophilus （with different dose）

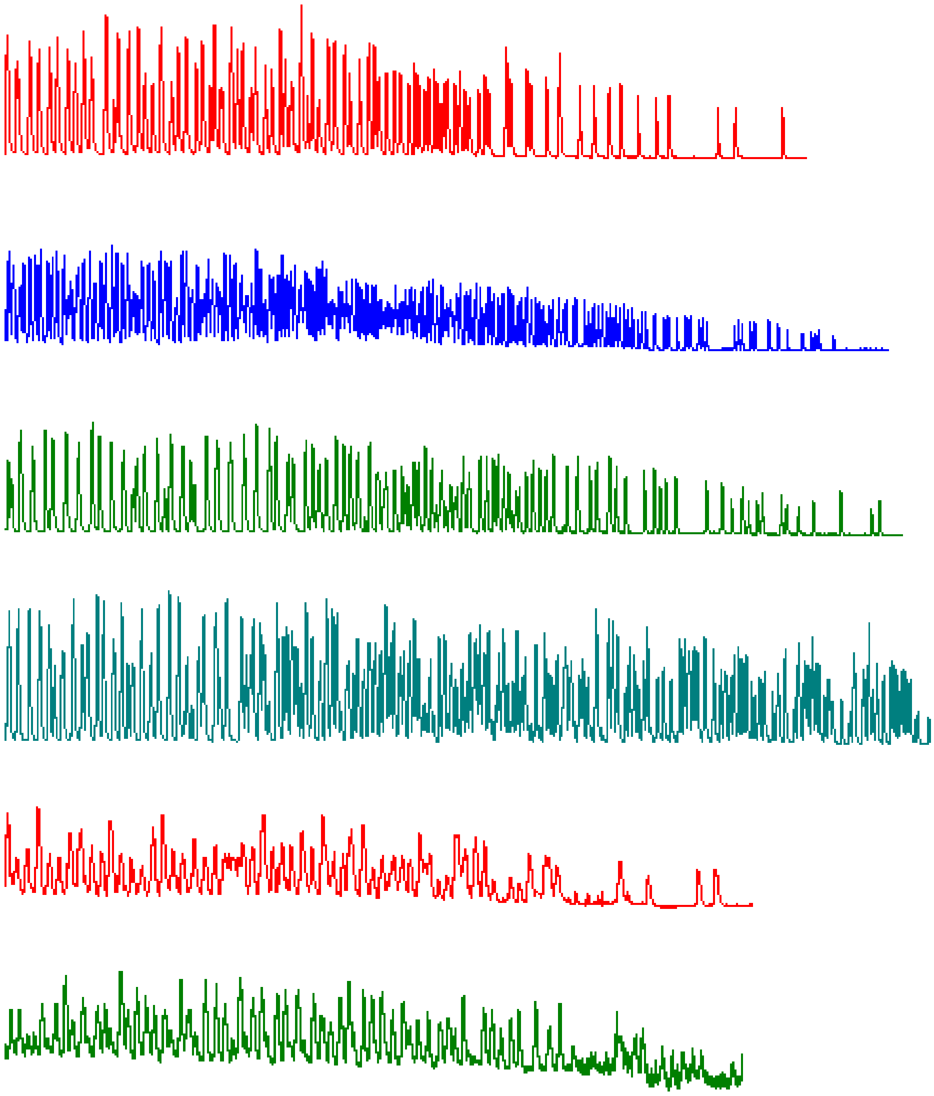

Entire living bacteria of Enterococcus faecalis （with different concentration）

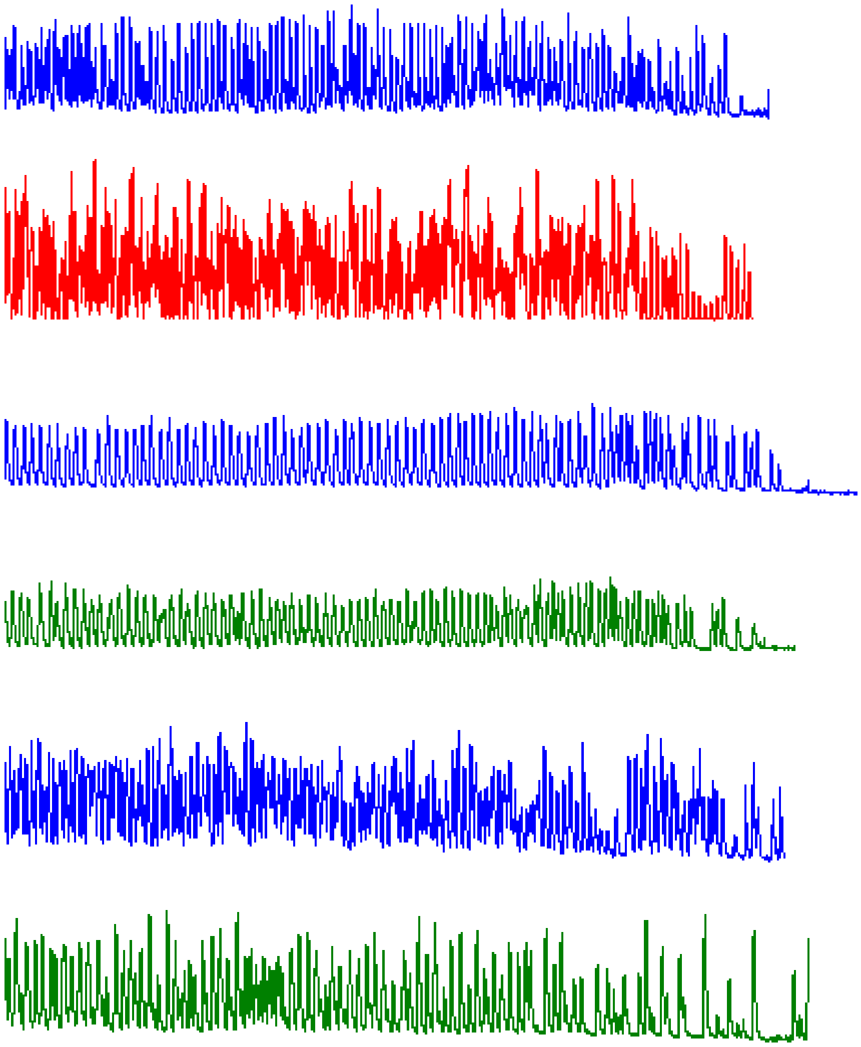

Cell-free supernatant of Enterococcus faecalis （with different dose）

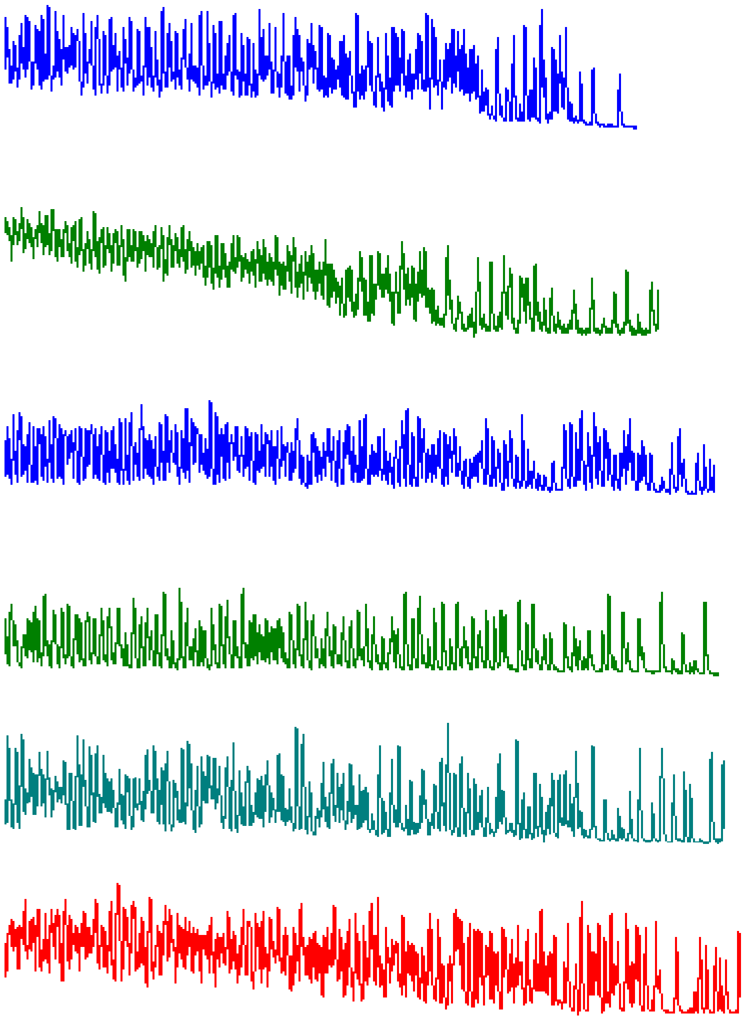

Crude extracts of Enterococcus faecalis（with different dose）

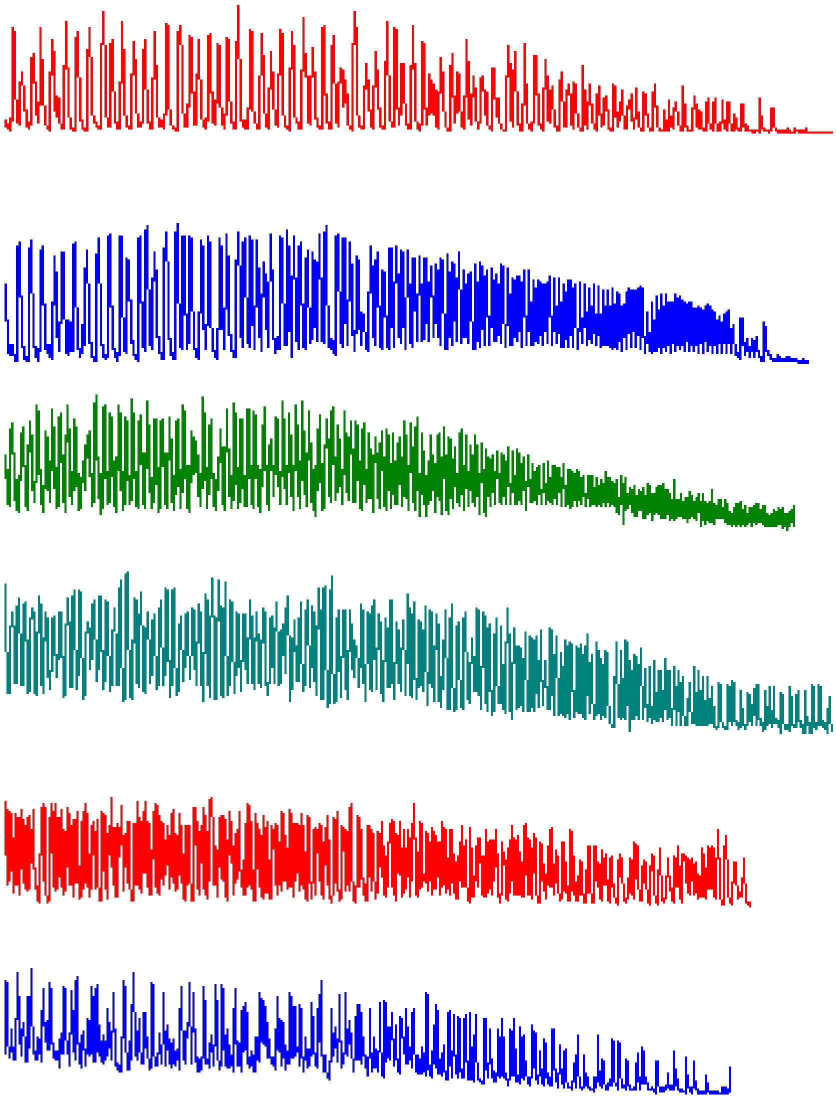

MRS：（with different dose）

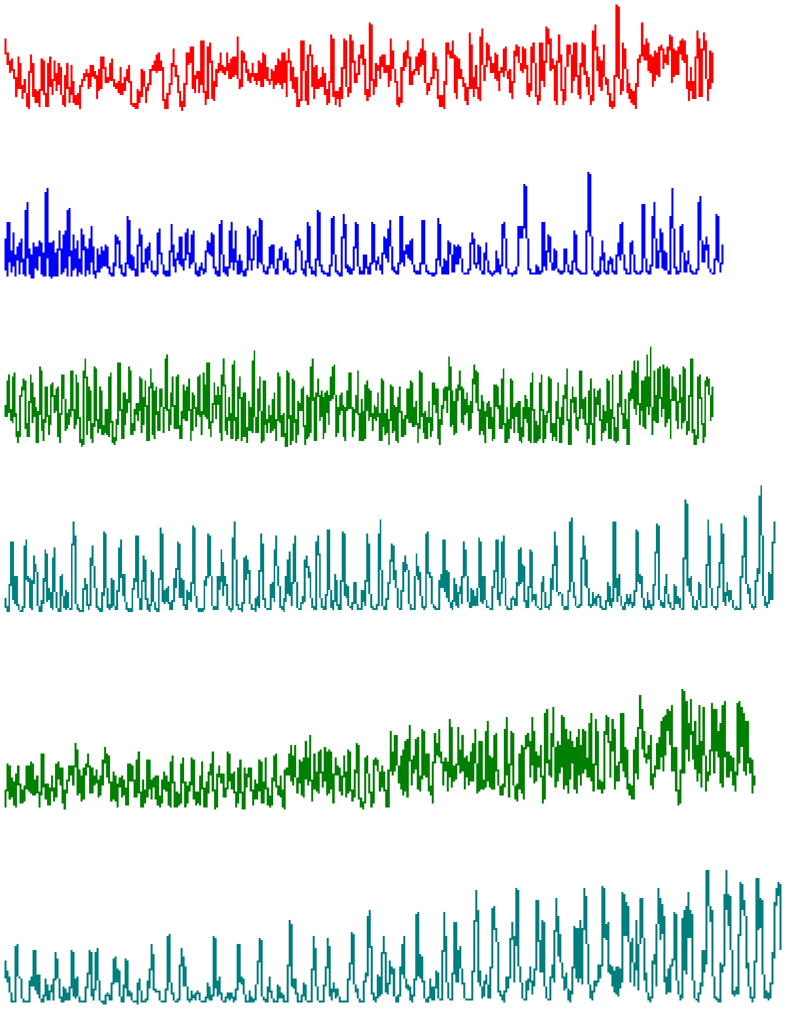

Krebs （with different dose）

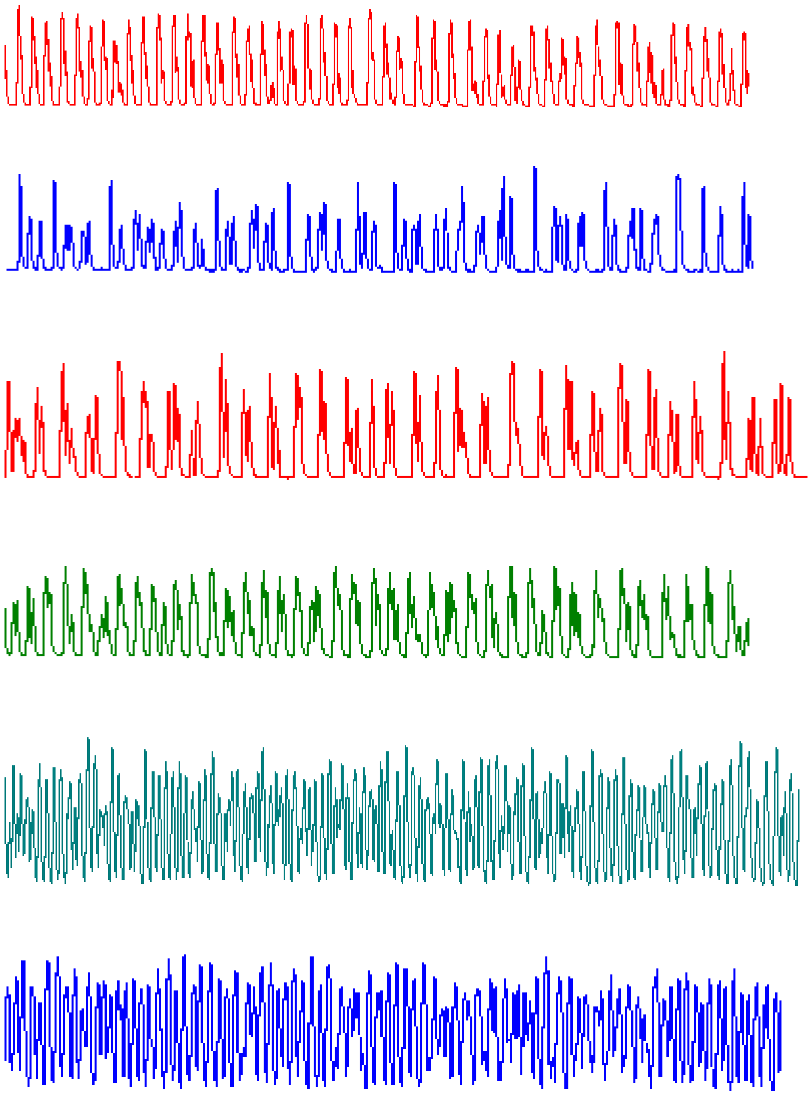

L-NNA pretreatment of Bifidobacterium longum （with different dose）

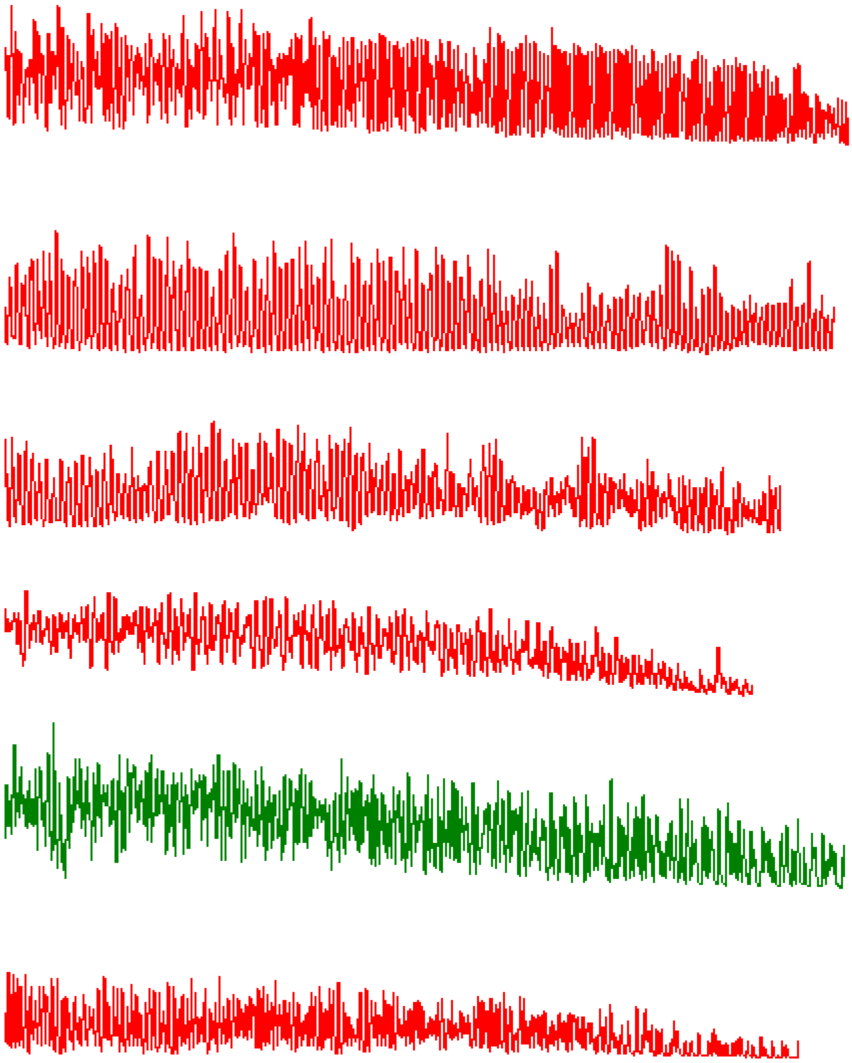

L-NNA pretreatment of Lactobacillus acidophilus （with different dose）

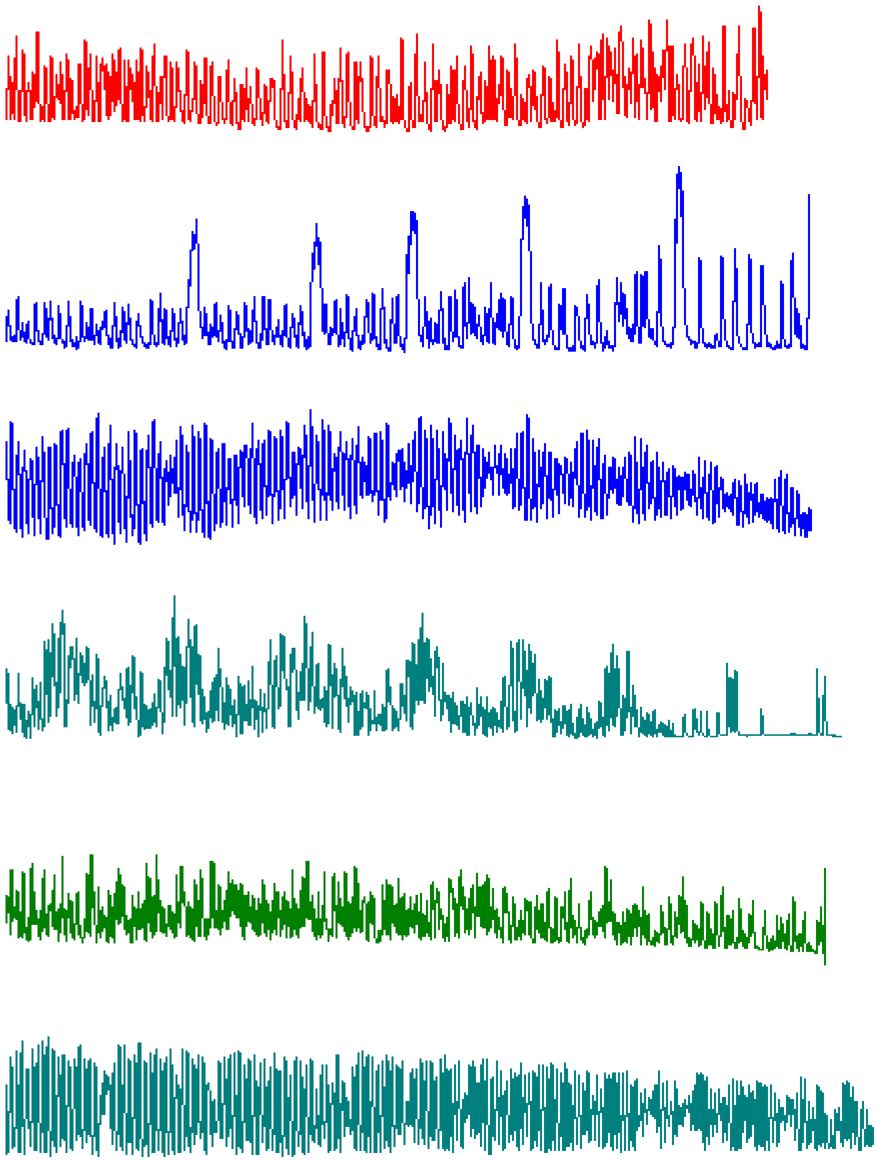

L-NNA pretreatment of Streptococcus themophilus （with different dose）

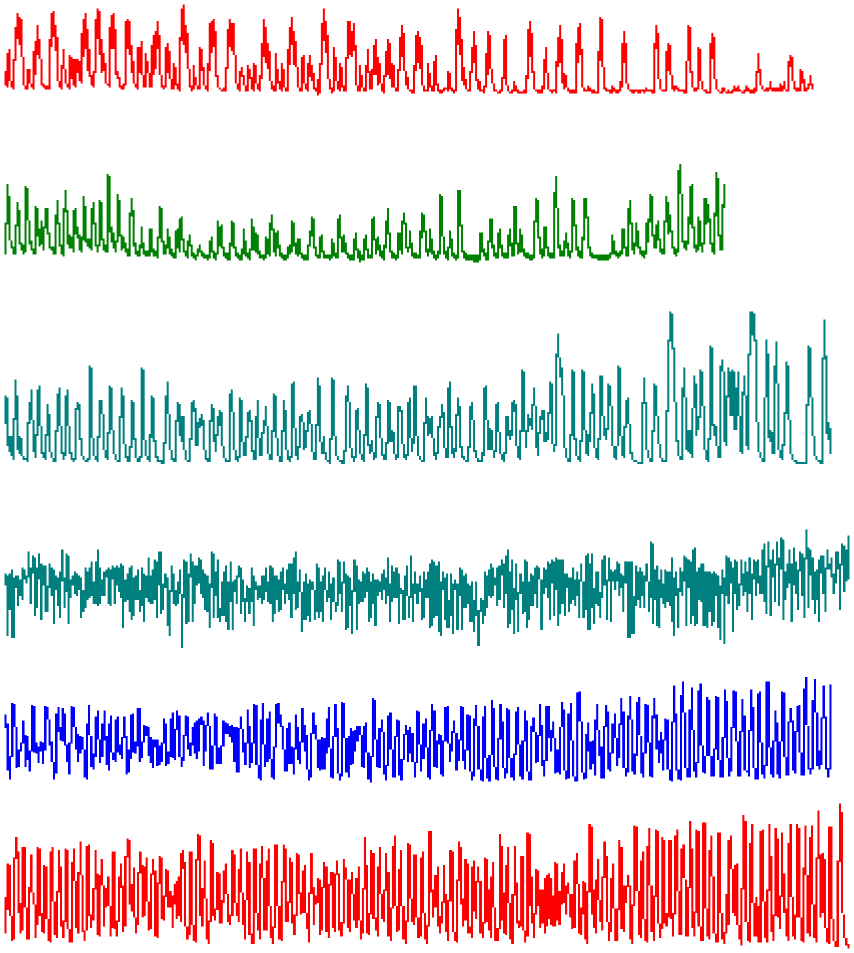

L-NNA pretreatment of Streptococcus themophilus （with different dose）

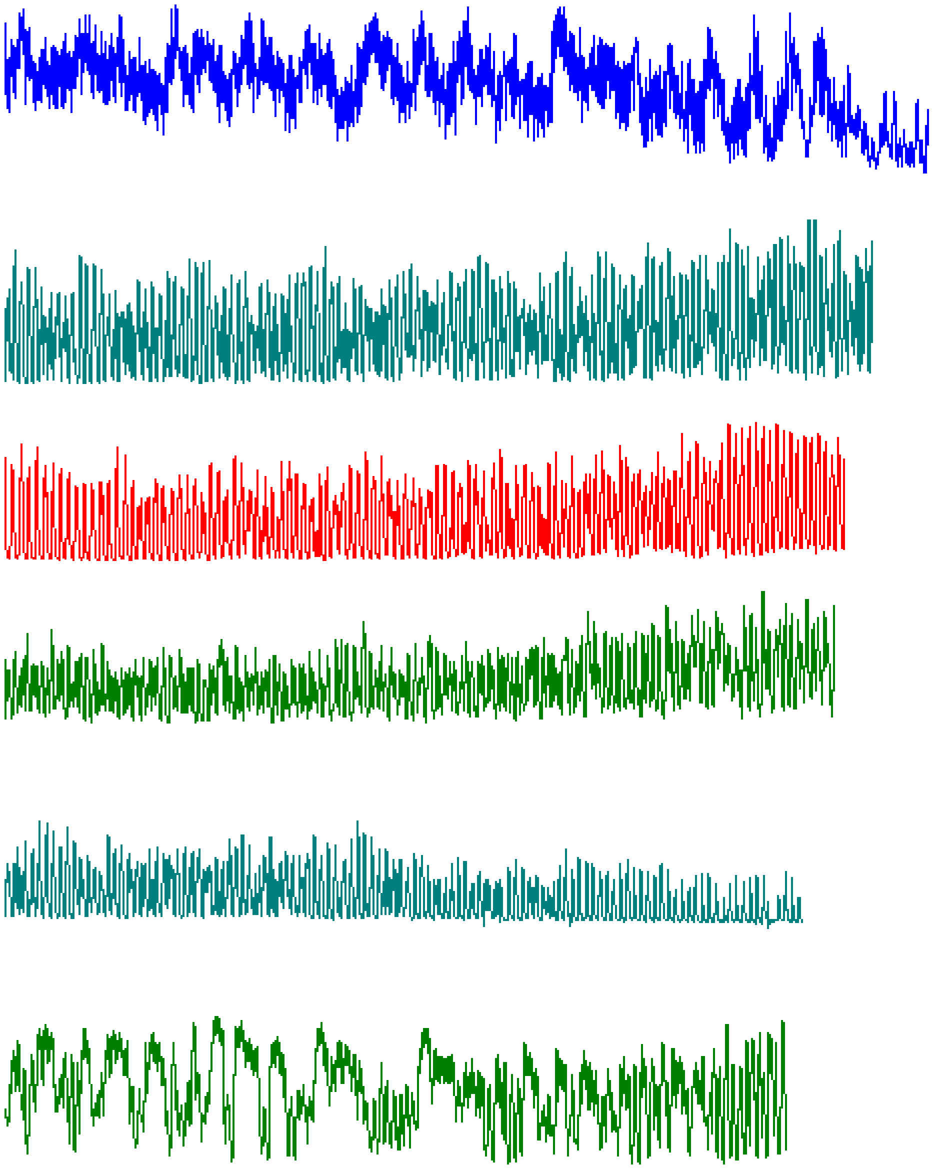

Supplement: S2 File — (PDF) [file pone.0189257.s002.pdf]
